# “I don’t know whether I really have choices”: a multiperspective and longitudinal exploration of healthcare choices of older adults with multimorbidity

**DOI:** 10.1093/geront/gnag216

**Published:** 2026-09-08

**Authors:** Thessa W Thölking, Yvonne Engels, Yvonne Schoon, Els J van Wijngaarden

**Affiliations:** Department of Anesthesiology, Pain and Palliative Medicine, Radboud University Medical Center, Nijmegen, The Netherlands; Department of Anesthesiology, Pain and Palliative Medicine, Radboud University Medical Center, Nijmegen, The Netherlands; Department of Geriatrics, Radboud University Medical Center, Nijmegen, The Netherlands; Department of Anesthesiology, Pain and Palliative Medicine, Radboud University Medical Center, Nijmegen, The Netherlands

**Keywords:** Phenomenology, Ethnography, Decision-making, Relationality, End-of-life

## Abstract

**Background and Objectives:**

As the prevalence of multimorbidity among older adults is rising, challenges regarding their end-of-life healthcare choices are increasingly relevant. To address knowledge gaps regarding decision-making, we explored how older adults with multimorbidity, their relatives and involved healthcare professionals (HCPs) experienced end-of-life healthcare choices, how their experiences related to each other and how these choices occurred in interactions between them over time.

**Research Design and Methods:**

We conducted a multiperspective, longitudinal, interview and observation study with a phenomenological and ethnographic approach. We followed nine 6-month patient trajectories with 11 relatives and 58 HCPs and conducted 73 observations of patient-healthcare professional interactions and 63 interviews.

**Results:**

The meaning of end-of-life healthcare choices of older people with multimorbidity was essentially understood as an ongoing, entangled, existential, and relational process, shaped by, yet also transcending, healthcare contexts. This process was characterized by four tensions: (i) the experience of not having a choice, but making decisions nonetheless; (ii) the existential experience and the medical gaze; (iii) the importance of relatives and their limited role in healthcare; and (iv) navigating fragmented healthcare with interrelated conditions.

**Discussion and Implications:**

HCPs’ limited insight into patients’ and relatives’ personal, existential perspectives and relational contexts calls for a better exploration of their lifeworld to ensure the best possible alignment of healthcare decisions with patients’ and relatives’ needs and values. Older adults with multimorbidity are inadequately supported by fragmented healthcare systems, and efforts to improve this should consider existential, relational, and systemic factors.

## Background and Objectives

The number of older adults with multimorbidity—mostly defined as the co-occurrence of two or more chronic conditions—is increasing globally, concerning at least two thirds of the population aged above 65 (Skou et al., 2022). Due to their single-disease focus, healthcare systems are mostly ill-equipped to offer integrated care to this population. Therefore, the rise of the prevalence of multimorbidity has been recognized as a substantial challenge for healthcare (Skou et al., 2022; The Plos Medicine Editors, 2023; Whitty et al., 2020).

Addressing the needs of older adults with multimorbidity is particularly pressing when it comes to decision-making in the end-of-life phase, as (i) clinical guidelines offer limited guidance (Boyd et al., 2019); (ii) healthcare decisions are ill-coordinated between various healthcare professionals (HCPs) (McGilton et al., 2018); (iii) disease trajectories are subject to continual change, and prognoses are unpredictable and uncertain (Etkind et al., 2022; Murray et al., 2002, 2017); and (iv) conversations about end-of-life topics are frequently avoided between patients, relatives, and HCPs (Sallnow et al., 2022).

Despite these challenges in decision-making, multimorbidity and the perspectives of older adults with multimorbidity and their relatives have been understudied (Mason et al., 2016). Moreover, while patient trajectories are dynamic and subject to change, most studies in healthcare remain cross-sectional (Murray et al., 2009). Similarly, although patients never exist in a social vacuum and healthcare decision-making is increasingly understood as a relational process (Gómez-Vírseda et al., 2019), multiperspective research is underutilized (Kendall et al., 2009; Vogl et al., 2018).

To address these gaps, we conducted a study with a wide scope on end-of-life healthcare choices of older adults with multimorbidity. Healthcare choices were understood broadly as any choices faced or decisions made regarding patients’ chronic conditions, overall health, or (daily) healthcare. Similarly, end-of-life was interpreted expansively as the phase in which patients were “at risk of deteriorating and dying” (Highet et al., 2014, p. 285). Consequently, healthcare choices made within this phase were considered end-of-life healthcare choices. Our aim was to explore how older adults with multimorbidity, their relatives and involved HCPs experience end-of-life healthcare choices, how their experiences relate to each other and how these choices occur in interactions between any of them over time.

## Research design and methods

### Research approach and study design

In order to gain in-depth insights into the complexity of the phenomenon end-of-life healthcare choices of older adults with multimorbidity and to understand how this experience is shaped by its social and institutional context, we combined a multiperspective and longitudinal design with two qualitative approaches: phenomenology and ethnography (Slatman, 2014).

The phenomenological reflective lifeworld approach allowed us to explore participants lifeworld and understand how the phenomenon under study was experienced (Dahlberg et al., 2008). In addition, a focused ethnographic approach was undertaken to contextualize the participants’ experiences within their social environment (Knoblauch, 2005; O’Reilly, 2012). As end-of-life healthcare choices are processes that do not occur in a single moment in time and involve multiple actors (Kendall et al., 2009; Murray et al., 2009), we applied a longitudinal and multiperspective study design (Vogl et al., 2018). In our design, we followed patient trajectories within (various) healthcare (institutions) by observing patients’ interactions with HCPs during a 6-month period and collecting reflections of participants by holding (in)formal interviews. More details on the methodology following the consolidated criteria for reporting qualitative research (COREQ) guidelines can be found in Supplementary material 1 (Tong et al., 2007).

### Study setting and healthcare context

This study was undertaken in the context of the resource-rich Dutch healthcare system in which healthcare needs are relatively well-met (Kroneman et al., 2025). In the Netherlands, essential medical care, including primary, hospital and mental health care, as well as district nursing, medications and medical aids are funded through a government determined basic health insurance package that is covered by mandatory health insurance (Kroneman et al., 2025; Ministry of Public Health Welfare and Sport, 2016). Out-of-pocket payments are generally not required when accessing healthcare or receiving treatment, apart from a compulsory annual deductible of €385 and occasional co-payments for some specific types of care (Kroneman et al., 2025; Ministry of Public Health Welfare and Sport, 2016). Residents are eligible for 24-hr home or institutional care financed nationally through income tax and additional personal income-dependent compensations (Ministry of Public Health Welfare and Sport, 2016). Altogether, out-of-pocket payments contribute less than 10% of total health spendings in the Netherlands (Kroneman et al., 2025).

### Participants and recruitment

We followed 9 patient trajectories in which we also included 11 relatives and 58 HCPs. One trajectory was limited to 3 months due to the patient’s death. The study was pre-registered at Open Science Framework (https://osf.io/xdvjs). The medical-ethical review committee (Nijmegen, dossier 2023-16433) reviewed the study and determined it was not subject to the Dutch Medical Research Involving Human Subject Act (WMO). Participants were recruited from a university medical center in a mid-sized city in the Netherlands and various general practices in the same city and surrounding towns. Specialists from varying specialties and general practitioners from different municipalities were invited to propose one patient for participation in the study. Purposive sampling was applied to seek diversity in sex, types of chronic diseases, different types of involved HCPs, education level, and socio-economic status (O’Reilly, 2012). Persons accompanying patients to healthcare appointments were asked to participate in the study. If patients attended appointments alone, they were asked to nominate one person that was important to them in healthcare decision-making. Whilst not required, these persons were all relatives of patients. Prior to healthcare appointments, the respective HCPs were invited to participate in the study.

Inclusion criteria for patients were: (i) age ≥75 years; (ii) presence of three or more chronic conditions; (iii) the including physician answered “no” to the so-called “surprise question” (would I be surprised if this patient were to die within 12 months?) (Romo & Lynn, 2017); (iv) involvement in and mental competency for making healthcare decisions; and (v) ability to complete an interview in Dutch or English. To focus on non-cancer chronic conditions as these have traditionally received less attention (Murray et al., 2002), patients were excluded if cancer was their primary health problem. We chose to include patients with at least three chronic conditions instead of only two to increase the likelihood of capturing experiences related to the intersection of having multiple chronic conditions.

### Data collection

Data were collected by researcher T.W.T. through observing healthcare interactions, conducting (in)formal interviews with participants, and reviewing electronic patient files.

During a 6-month period, the researcher accompanied patients, if applicable together with relatives, to various healthcare appointments. All scheduled interactions with HCPs that were expected to (partly) contain dialogue with patients were considered eligible. Appointments were mainly outpatient visits with physicians and (specialized) nurses, but also included clinical rounds, inpatient family meetings, allied health professional treatment sessions and home visits (see Supplementary material 2 for more details on observations). Before and after appointments, the researcher held (joint) informal interviews in the waiting rooms about how things were going, expectations beforehand and immediate reflections afterwards. During the consultation, she made an audio-recording and initial observational fieldnotes, which were later completed (Emerson et al., 2011; O’Reilly, 2012) (see Supplementary material 3 for the observation guide). During telephone consultations, the researcher joined the HCP to make an audio-recording. In case the researcher was unable to attend an appointment, patients or HCPs were invited to make an audio-recording for the researcher. In total 73 appointments were included, with a total of 31 hr of audio-recorded data.

At the beginning and end of the 6-month study period, patients and (most) relatives were invited for a formal interview about their experiences of healthcare decision-making. These interviews were semi-structured, phenomenological, in-depth interviews (Dahlberg et al., 2008). At the start of the interview, participants were invited to reflect on their experience of choices (of their relative) in healthcare in general. The researcher followed participants’ narratives and asked explicating questions to further unravel participants’ experiences. Particularly, experiences of decision-making, interactions with HCPs, previously observed interactions, and the end-of-life were explored (see Supplementary material 4 for the interview guide). Participants were mainly interviewed in their home, but incidentally in the hospital or by phone. In total, 18 patient interviews (mean duration 77 min) and 17 relative interviews (mean duration 59 min) were held.

After interactions that were relevant regarding decision-making or at the end of the 6-month observation period, HCPs that were involved with decision-making were invited for an interview, with incidental serial interviews in case of ongoing decision-making. Structurally, the interviews were similar to the patient and relative interviews. They started with inviting the HCP to tell something about their experiences of decision-making specifically with the participating patient and relative(s). HCP interviews were conducted in participants’ workplaces or through a video (and incidental telephone) call. Thirty HCP interviews were held (mean duration 30 min) (see Supplementary material 5 for more details on interviews).

After the study period, HCP notes in patient files and written communications to other HCPs were obtained.

### Data analysis

Data analysis was grounded in a phenomenological approach (Dahlberg et al., 2008). We developed an analytical guide to integrate and manage the fast volumes and different types of data from various perspectives and timepoints compatible with phenomenological epistemology (Antonides et al., 2026). The analysis started with familiarization with the data and identification of meaningful parts. In these phases, parts of the data and evolving insights were regularly discussed in the research team. The research team consisted of researchers with backgrounds in medicine, care ethics, geriatrics, health science, medical anthropology, psychology, and spiritual care. To condense and integrate data from within patient trajectories, we drafted long, typically 50–70-page narratives per patient trajectory from the identified meaningful parts. In these narratives, data were arranged thematically, bringing together parts of interviews, observations, and patient files on the same topics and/or events. Two narratives were constructed jointly by researchers S.v.F. and T.W.T., after which T.W.T. drafted the remaining seven narratives. The narratives were discussed within the research team to refine the narratives and to discuss analytical insights. Going forward, the narratives were used as the basis of further phenomenological analysis to identify and cluster meaning units. This phase was assisted by coding meaning units in Atlas.ti (version 24). The largest narrative was coded independently and discussed by Y.E. and T.W.T., whereafter T.W.T. coded the remaining narratives. The patterns of meaning that emerged from clustering were further developed into themes that formed the main constituents and essence of the phenomenon under study, which were further evolved through reflective writing and team discussions. Participant citations reported in the results were taken from the narratives and are therefore composite citations, consisting of (partial) quotes by participants made in different (parts of) interviews, observations, or from fieldnotes (Crowther et al., 2017). To reduce the identifiability of participants amongst each other, some details about participants were slightly altered.

## Results

The mean age of the nine patients was 81 ± 5 years, and four were women. They reported having between 6 and 10 chronic conditions. Five patients lived together with their partner, whilst three lived alone and one lived in a long-term care facility. Six of the eleven relatives were spouses, the other were children. Their main age was 67 ± 11 years and seven were women. Thirty of the 58 HCPs were physicians. Other HCPs included nurses, general practitioner assistants, physiotherapists, and other allied health professionals. The mean age of HCPs was 43 ± 12 years and 39 were women. The length of their relationship to the patient was a median of 0.5 years (IQR 0–5). Further demographic characteristics of participants and details of the patient trajectories are described in Tables 1 and 2. The patient trajectories are described in more detail in Supplementary material 6. Within the patient trajectories, a large variety of types of healthcare choices was observed, e.g., choices about (daily) care, lifestyle, mobility, access to healthcare, patient-HCP relationship, diagnostics, treatments, and treatment limitations (see Supplementary material 7 for details).

**Table 1:** Participant demographic characteristics.^a^

Category	Patients, *n* = 9	Relatives, *n* = 11	HCPs, *n* = 58
**Age in years**	81 ± 5	67 ± 11	43 ± 12
**Female sex**	4 (44%)	7 (64%)	39 (67%)
**Country of birth**			
The Netherlands	8 (89%)	7 (64%)	58 (100%)
Western European country	1 (11%)	1 (9%)	–
Western Asian country	–	1 (9%)	–
Northern American country	–	2 (18%)	–
**Religious affiliation**			
Catholic	6 (67%)	8 (73%)	18 (31%)
None	3 (33%)	2 (18%)	36 (62%)
Jewish	–	1 (9%)	–
Protestant	–	–	3 (5%)
Islam	–	–	1 (2%)
**Marital status**			
Married	6 (67%)		
Widowed	3 (33%)		
**Children**			
0	3 (33%)		
1–2	4 (44%)		
3	2 (22%)		
**Highest level of education**			
Primary school with uncompleted further education	1 (11%)	1 (9%)	
(pre-)vocational secondary education	1 (11%)	1 (9%)	
Secondary vocational education	3 (11%)	2 (18%)	
University entrance level	1 (11%)	1 (9%)	
University/higher professional education	3 (11%)	6 (55%)	
**Most important (former) occupation**			
Education	2 (22%)	4 (36%)	
Administrative	3 (33%)	1 (9%)	
Healthcare	1 (11%)	2 (18%)	
Other^b^	3 (33%)	3 (27%)	
None	–	1 (9%)	
**Living situation**			
Independent, with partner	5 (56%)		
Independent, alone	3 (33%)		
Long-term care facility	1 (11%)		
**Clinical Frailty Score (1–9)**	4.9 ± 0.9		
**EQ VAS^c^ (0–100)**	69 ± 15	66 ± 25	
**EQ-5D-5L^d^ Mobility (1–5)**	3.3 ± 0.7		
**EQ-5D-5L^d^ Self-care (1–5)**	1.8 ± 1.1		
**EQ-5D-5L^d^ Usual activities (1–5)**	2.4 ± 0.7		
**EQ-5D-5L^d^ Pain/discomfort (1–5)**	2.9 ± 1.2		
**EQ-5D-5L^d^ Anxiety/depression (1–5)**	1.8 ± 0.8		
**Length of relationship to patient in years**		55 ± 8	Median 0.5 (IQR 0–5)
**Type of relationship to patient**			
Spouse		6 (55%)	
Child		5 (45%)	
**Caregiver Strain Index (0–13)**		5.6 ± 3.5	
**Type of healthcare professional**			
Medical specialist			16 (27%)
Nurse^e^			12 (21%)
Resident			8 (14%)
General practitioner			7 (12%)
Physiotherapist/exercise therapist			6 (10%)
General practitioner assistant			3 (5%)
Nurse practitioner/physician assistant			3 (5%)
Other allied health professionals			3 (5%)
**Work setting**			
Primary care			22 (38%)
Hospital			7 (12%)
University medical center			29 (50%)
**Work experience in current occupation in years**			13 ± 11

*Note.* HCPs = healthcare professionals.

a Characteristics are represented as “mean ± standard deviation” or “*n* (%)” unless otherwise specified.

b Other patient occupations included managerial, analyst, and construction. Other relative occupations included journalism, culinary, and technician.

c EuroQoL visual analogue scale.

d EuroQoL-5 dimension-5 levels.

e Includes nine registered nurses working in hospitals, two district nurses working in home care and one nursing associate working in a long-term care facility.

**Table 2:** Details of patient trajectories.

Patient sex & age	Health conditions^a^	Included relatives	No. of HCP^b^	No. of observations
Male, 87 years	Kidney failure, diabetes, coronary artery disease, atrial fibrillation, diabetic retinopathy, and restless legs syndrome.	Wife, daughter, and son	10	8
Male, 78 years	Heart failure, atrial fibrillation, cervical and lumbar spinal cord stenosis, chronic pain, prostate cancer, gout, osteoarthritis, aortic ectasia, and hypercholesterolemia.	Wife	18	30
Male, 75 years	Heart failure, hypertension, peripheral arterial disease, bipolar disorder, urine incontinence, and osteoarthritis.	Wife	4	3
Male, 80 years	Chronic obstructive pulmonary disease, atrial flutter, kyphoscoliosis, osteoarthritis, osteoporosis, and hypercholesterolemia.	Wife	6	8
Female, 92 years	Heart failure, hypertension, peripheral neuropathy, restless legs syndrome, urine incontinence, and hypercholesterolemia.	Daughter	6	5
Female, 77 years	Polyarthrosis with multiple joint replacements, chronic pain, pelvic organ prolapse, polymyalgia rheumatica, chronic constipation, thrombotic disorder, hemochromatosis, hypertension, osteoporosis, and xerosis.	Husband	4	6
Female, 81 years	Heart failure, hypertension, cardiac arrhythmia, chronic pain, urine incontinence, asthma, rheumatic disorder, glaucoma, and eczema.	Husband	5	5
Male, 80 years	Cardiac arrhythmia, hypertension, hypercholesterolemia, partial deafness, insomnia, and anemia.	None	2	2
Female, 82 years	Asthma, coronary artery disease, hypertension, renal insufficiency, skin cancer, partial deafness, thyroid cyst, hay fever, and dust mite allergy.	Daughter and son	5	6

*Note.* HCP = healthcare professional.

a As reported by the patient at the start of study. Incidentally, additional conditions were added by the researcher if they were prominent during the observations (supported by patient files) or developed during the study.

b Two healthcare professionals were part of two patient trajectories.

In this study, the meaning of the phenomenon “end-of-life healthcare choices of older people with multimorbidity” was essentially understood as: an ongoing, entangled, existential, and relational process, shaped by, yet also transcending, healthcare contexts. As patients’ experiences unfolded temporally, choices appeared as processes with no clear start and end point. Choices were shaped by patients’ previous (healthcare) experiences, identities, and values that far preceded being faced with a particular decision. And after decisions were made, these remained present, as they could sometimes still be subject to change, and their outcomes were relevant to patients’ (daily) lives and continued to factor into future decisions. Due to their multimorbidity, patients continuously faced entangled healthcare problems that called for—sometimes contradicting—choices. The complexity of these choices was often heightened by the uncertainty of their outcomes. The choices were approached from different perspectives by patients, relatives, and HCPs. The interdependencies and power dynamics between these different actors resulted in tensions in the decision-making process. The healthcare system formed a framework that shaped the way in which decisions could be made. Ultimately, the healthcare choices impacted patients’ and relatives’ (daily) lives existentially, therefore the choice processes transcended beyond healthcare.

Four tensions were identified that constitute the essence of the phenomenon healthcare choices of older adults: (i) the experience of not having a choice, but making decisions nonetheless; (ii) the existential experience and the medical gaze; (iii) the importance of relatives and their limited role in healthcare; and (iv) navigating fragmented healthcare with interrelated conditions. For each theme, an illustrating portrait was crafted from one patient trajectory.

### The tension between the experience of not having a choice, but making decisions nonetheless

#### Illustrative portrait: “if the body won’t, it won’t”

Vernon felt that he had no choices to make about his healthcare. About the decision to start dialysis 5 years ago during acute illness, he said: “*The physicians said, ‘you have to undergo dialysis.’ So that decision was, so to speak, imposed from the outside. If I don’t have a choice, I’ll have to accept it. I wasn’t keen on going to heaven yet.*” During his dialysis, Vernon faced institutional rules and medical protocols. When the dialysis kitchen staff informed him that eating a fruit salad wasn’t allowed at that time of day, he politely replied: “*The rules are the rules.*” But after they had left, he turned to me, sighing: “*The holy rules.*” The frequent repeated measuring of his blood pressure drove him crazy: “*The first time I don’t mind, but the second time, I really can’t stand that. And then the third, fourth, fifth time. That’s just too crazy to be true. Then I protest.*” But his protests were in vain: “*Nothing happens. It will be measured again. Only a bit later. Unfortunately, by and large we don’t have too much to say.*” His subtle protest was to talk during measurements, telling me: “*We will not allow ourselves to be tyrannized.*” Vernon had a clear goal for his healthcare choices: “*To undergo as little change as possible.*” He tried to minimize the impact of his diseases on his daily life: “*What must be done, must be done. I’ll do what’s necessary, okay, but nothing more than that.*” In line with this philosophy, I observed Vernon to make multiple decisions for his healthcare, including opting against cardiac catheterization and compression stockings that entailed daily home care visits. Regardless, Vernon felt that he had made no choices: “*What choices do I have? Those choices are imposed from the outside. The fact that I have kidney problems, what can I do about that? Everything slowly breaks down. If the body won’t cooperate, what can I do against that? It happens to you. My own contribution is that I accept it as best as I can.*” Vernon also felt that his choice freedom was limited by his lack of medical knowledge: “*I don’t know whether you really have that many choices. You ask, ‘what is good medication’, and then that’s what you want to have. What medication I need, I can’t say. I’m not a professional. What they need to do is something that falls more within their judgment than mine.*”

#### Description of tension

During observations, patients were seen to be making multiple decisions regarding their healthcare. When asked about their experiences of choice within the healthcare system, however, patients mostly responded not to experience having choice. Not experiencing choice was a layered experience, emerging from a diverse range of underlying experiences. Firstly, patients shared different experiences on a personal level that negated a sense of choice. Secondly, they expressed dependencies on HCPs that limited their experience of choice. Finally, patients sometimes described that they felt they were not making choices, as choices seemed to be dictated by the healthcare system. These choices included restricted options for patients in choosing their healthcare center or specific HCP to receive care from, unavailable care due to staff shortages, or waiting lists.

On a personal level, various experiences were underlying to the experience of “not choosing.” Some patients stated that their health and illness was something that just “happened” to them or that they had no choices to make as they were physically dependent to accomplish their activities of daily living. In other instances, patients expressed that choice options were too risky to be viable or that they had no realistic (other) options to choose from if they wanted to survive or keep functioning in their daily life. Similarly, the severity of physical symptoms could also seem to necessitate a certain choice:

*The burden. The discomfort. I was in a great deal of pain. And then there’s a proposal to do a pain treatment. You grab a proposal like that with both hands. I don’t think there was a single moment when I felt I had a choice to make. Everything was simply dictated by the seriousness of the situation.* [Otis]

In interviews, patients frequently described that they felt dependent on their HCPs. This included patients sharing that they themselves did not have the knowledge to realize or understand what their options were. Similarly, they expressed feeling dependent on the professional expertise and judgement for decisions, but also to navigate the healthcare system and to get access to care, as HCPs mostly decided which options were on the table. Patients who were dependent on HCPs for activities of daily living described that this contributed to a sense of powerlessness. Edward shared a situation in which he experienced no choice when he was washed in the morning in the care institution where he lived by substitute HCPs that he did not know and that he felt uncomfortable with:

*I have to make that choice. I have to be cared for. Suddenly they’re touching you, grabbing you, washing you, and so on. I found it painful to have people all over me whom I knew nothing about. Who just suddenly start. And that happens a lot in healthcare. I found that very unpleasant. That’s the power of the institution. I’m just a client; I don’t have that power. If I say no, I won’t be washed.*

Patients often relied on their HCPs that they’d know “what’s best” and did what was necessary:

*You go to the doctor and you say what’s wrong with you. Then you get an answer, or a little pill. I don’t see that many options to choose from. I mean, the doctors know what they’re doing. Well-intentioned, all of it*. [Simon]

Observations revealed that patients were regularly not involved in decision-making or not given choice by HCPs. In some situations, HCP did not inform patients of—often technical—decisions that were made in the background during treatment. More visible to patients, HCP made—mostly protocol driven—decisions about medications, interventions, or institutional admission or discharge without involving patient perspectives. Not being offered a choice could lead to patients feeling frustrated or not taken seriously but was also often taken for granted or expected.

While patients predominantly expressed not to experience choice, and multiple observations revealed a similar lack of choice, other observations and parts of patients’ accounts showed patients in a position of agency. For instance, patients frequently chose to make effort for their health through lifestyle interventions, staying active, seeking the best possible healthcare, educating themselves on their health and healthcare, quitting hazardous and addictive medications, monitoring their symptoms closely and using assistive devices.

Patients also frequently made requests to HCPs. These requests varied and included requests for expediting care, additional appointments, referrals, medication changes, (diagnostic) procedures or operations, preferences about how care was provided, treatment limitations, or euthanasia. In multiple instances, patients decided to stop or interrupt treatments they were receiving, due to seeing no purpose for those treatments or experiencing side effects. Or patients decided to refrain from treatment options suggested to them by HCPs:

*Up until last year, I tried everything she* [physician] *suggested. I went along with everything, just to try to make things go as well as possible. And after all those years it still didn’t work. None of it really helps anymore, and I don’t have the energy for it all anymore either, all the bells and whistles, all those bladder irrigations. I just can’t bring myself to do it anymore, so I’m trying to accept it the way it is.* [Margaret]

Commonly, decision-making was observed as HCPs explaining one or more options to patients and consequently letting patients decide if they consent with the one option or between the multiple options. In interviews, some HCPs expressed that they explicitly let patients make their own decisions. They mentioned respecting patient autonomy and preferences, ensuring a good and trusting HCP-patient relationship or patient compliance, and contributing to patients feeling heard and taken seriously as reasons for this approach.

Walter was the most notable exception of the population, consistently expressing that he experienced choice. Nonetheless, protocol-driven decisions were also observed in his trajectory and his general practitioner reported that when being faced with a complicated decision whether to admit Walter to the hospital or not: *“He actually preferred that I made the decision*.” [HCP36]

### The tension between the existential experience and the medical gaze

#### Illustrative portrait: “I am completely afloat”

Ruth was very attached to her house, which held many memories of her children and her late husband. Over time, she experienced increasing problems with her mobility and energy levels: “*I’ve become so incredibly dependent. I had never imagined that I would become so helpless that I should be able to call someone all day and all night if something happened.*” Ruth shared an experience of getting stuck on the side of her bed: “*I couldn’t get up. I had nothing to hold on to. I kept thinking how on God’s earth am I supposed to get back into bed? I sat there for quite a while. Then I had an idea. It took a long time. Eventually, I pulled the duvet over me and lay there crying for fifteen minutes, out of sheer helplessness. That’s the thing. You feel utterly helpless. If I’m standing somewhere and my walker has rolled away, I honestly wouldn’t know how to get out of that situation*.” Ruth started having doubts whether she could stay in her beloved house: “*Six months ago, I couldn’t have imagined that. I’ve always said, ‘I’m going to die here.’*” She considered moving to a nursing home and felt strongly that it was her decision to make: “*I’ll have to make that choice, because I’ll have to live there. I wouldn’t want to burden anyone with that.*” But the burden of making the choice herself was a lot for Ruth: “*It upsets me that I have to think of everything and decide myself: what is the next step? What do I need to do? At the moment, nobody can think along with me and that causes me to panic.*” Facing these choices without her husband was hard on Ruth: “*I am grieving again. I’m missing my husband terribly. Sixty years you made all decisions together and now I have to figure it out all by myself.*” Recognizing her difficulties, the home care nurse organized a meeting with a palliative care nurse to discuss Ruth’s options. In this meeting, many practicalities were discussed pertaining different options for increased care at home. The meeting ended with a summary for Ruth by the nurse: “*Well, you were already looking into all of your options. Once you have an overview of your options, you’ll make your decision. And then we’ll look into what we can do to help you to realize that choice*.” Ruth reflected on this meeting: *“It wasn’t very helpful. Not that it wasn’t helpful at all, but ultimately, I have to decide for myself.*” Shortly after the meeting and at a peak of uncertainty, Ruth exclaimed, “*I am completely afloat,*” and decided to postpone her decision: “*I have decided not to make a decision right now, because I can’t do it. I just can’t. I know I’m just putting it off. But having made this decision, I do have more peace.*”

#### Description of tension

The healthcare choices patients were facing were often existential ones, as health problems limited the way in which patients could engage in activities, relationships, and ways of living that gave their life meaning and ultimately could be a threat to patients’ lives. Patients often shared worries and fears that were connected to these choices and faced uncertainties that they were sometimes trying to gain control over or that kept them occupied with doubts.

Patients were facing choices in changeable circumstances, as their health and mobility were dynamic, which meant that over time their choices sometimes changed too. Patients often anticipated (further) deterioration of their health and mobility in their choices, but sometimes they found themselves in situations they had not imagined. Frequently, previous healthcare experiences of patients themselves or relatives influenced how patients considered current and future choices, mostly as worst-case scenarios to avoid, but incidentally also as reassuring experiences providing trust.

A part of the HCPs had some insight into the existential dimension of their patients’ lifeworld. These were mainly HCPs with a long(er) history with the patients, HCPs from specialties with attention for this dimension, or HCPs that generally had longer appointment slots and therefore had more time to talk. These HCPs were sometimes aware of what gave meaning to patients’ lives, patients’ hopes and fears, their values, future goals, and preferences. Patient files, however, contained little references of such existential concerns.

Overall, HCPs mostly adopted a medical gaze in encounters with patients and conversations between them often had a (bio)medical, technical and/or pragmatic, solution-oriented focus. Oftentimes, HCPs limited conversations to the somatic domain:

*At that point, we mainly talked about physical complaints and how things were going, his* [patient] *anticoagulation, what we were going to do and the pain. But not on a higher level of ‘gosh, this is the second admission in such a short time, is everything alright?’ I didn’t discuss that with him*. [HCP5]

In these conversations, HCPs gave technical explanations that were not always understood by patients and relatives. The focus on practical solutions, instead of existential aspects, could leave patients insufficiently supported in difficult situations as illustrated in the portrait of Ruth.

In conversations with patients, as well as in their interviews, HCPs frequently talked in terms of “risks” and “managing patients’ expectations” when sharing their considerations. HCPs often commented on patients’ “capacity” and “competence” to oversee and understand things, often commending “realistic” patients: *“That does make him easier*” [HCP33]. When HCPs talked about patients’ “quality of life” in interviews, they often could not or only superficially explicate what this meant specifically to their patient. For instance, Vernon’s physician explained that she assumed he experienced quality of life because of: “*His kind of optimism, the way he came across. That he was cheerful*” [HCP5]. Vernon himself, however, commented on the toll that his situation took on him: “*My treatment is quite an ordeal that can make you feel very depressed from time to time.*” After Vernon’s death one HCP reflected on their limited insight into Vernon’s lifeworld:

*I’m realizing that more in this interview. You have the feeling that you know each other quite well. But now that I’m talking about it, I think, ‘well, there are some things I just don’t know’. I also never asked. We never discussed with him how he had envisioned his end. It’s actually quite strange that we never had this conversation with him.* [HCP3]

Patients sometimes made statements towards HCPs referring to an existential topic, but those cues were often missed and not explored further by HCPs. Similarly, topics regarding end-of-life, death, and dying remained largely undiscussed, also in situations when HCPs and patients had explicit considerations regarding this topic. When the end-of-life was discussed between patients and HCPs, it was mostly in the form of treatment limitations and prompted by an event like a (sudden) health deterioration, intervention, or treatment decision. Sometimes HCPs seemed to make statements to patients that implicitly referenced the patient’s mortality, and some HCPs shared with the researcher that they found this topic difficult to discuss. To a few HCPs, the topic seemed to come more naturally, and they could be observed casually weaving it into conversations about other topics. Some patients and relatives also expressed difficulties in discussing end-of-life topics. For many of them, however, the realization of an approaching death was top-of-mind. Only incidentally, HCPs reported trying to discuss end-of-life topics with patients and being met with resistance by patients and relatives.

### The tension between the importance of relatives and their limited role in healthcare

#### Illustrative portrait: “I think he’ll pretend for a long time that everything is going well”

Walter and Helen had been together for 60 years. Whilst Walter usually seemed to be resigned about his health, Helen worried extensively: “*I am very alert at all times, even at night. If I hear anything, I wake up immediately. Sometimes I sit up straight in bed and listen for a moment, is he still breathing?*” Helen always kept a close eye on Walter. When she left the house, she checked her phone regularly: “*If something happens, I’ll be home in no time.*” Helen knew Walter was a bit annoyed by her continuous inquiry of how he was doing: “*Once a day is enough, he says.*” When Walter’s condition worsened, Helen urged him to contact his doctor, but he refused: “S*he is worried because I won’t call immediately. But it’s my decision. I don’t want to call every two minutes or every two days. So as not to bother them with all sorts of nonsense. This doesn’t cause tensions. At least, not for me.*” Helen wished that HCPs would inquire into how she is doing: “*I’ve never heard from anyone: how are you? They just say that we’re doing well together. That’s true. But it would be nice if someone asks: how are you? Are you coping with everything?*” Walter’s cardiologist did not know much about Helen’s role, as she mainly held telephone consultations: *“I always have him on the line, and he always says that everything is fine.*” Walter’s pulmonologist had some more insight into Helen’s situation: “*The last time, she made it very clear that she was having a hard time, but that was actually the first time she had done so.*” Walter and Helen’s general practitioner had only talked superficially about the situation with Helen, as the general practitioner stated: “*Walter has told me a few times that she is worried and sometimes sleeps poorly. I’ve never talked with Helen about the disease, except for ‘well, are you aware that your husband is quite ill?’ She’ll confirm that, but we haven’t talked about anxiety or tensions.*” The general practitioner shared that he thought that Walter was not completely open in showing how he was doing: “*I imagine that he keeps up appearances as long as possible, because he really wants everything to be well. I think he would find it very upsetting if his wife gets anxious. So, I think he’ll pretend for a long time that everything is going very well. I often find it difficult to gauge how short of breath he really is. I don’t know if Walter is being completely honest about that. Because he always says that everything is fine.*”

#### Description of tension

The relatives in the study all had decades-long relations with each other and, especially between partners, their daily lives were intertwined. Due to their health problems, patients were often—in varying intensity—dependent on relatives for help and care in daily living, transport, and/or housekeeping, which was sometimes described as a burden by relatives: “*You really are ill together*” [Dorothy, wife]. Relatives frequently shared worries about how patients got on, their health status and potential death. Some also expressed feeling responsible for the patient’s health. Worries were often mutual, as patients also articulated worries about their relative’s health or about how relatives would cope if the patients themselves died. Relatives shaped patients’ considerations regarding healthcare choices by sharing their perspective, expectations or advise and frequently tried to convince patients of their perspective on choices. Patients often worried about overburdening their relatives and made decisions that contributed to a minimization of that burden.

In all patient trajectories, we found moments in which the needs of the patient and the relative were not aligned. Tensions could arise if relatives were (over)burdened by the situation. Similarly, tensions arose when patients did not agree with relatives’ worries or if patients and relatives disagreed about the care the patient needed. Patients and relatives alike sometimes shared that they felt that their perspective was not considered by the other: “*They call me stubborn. I think that’s a real shame. I think they’re really looking at it from only one side*” [Agatha].

Sometimes the misalignment of patients’ and relatives’ needs was limited to a subtle tension in the relationship, but often these tensions led to small and occasionally bigger conflicts. Patients and relatives frequently had difficulties communicating with each other. Some had difficulties in discussing existential topics with each other, whilst others had difficulties in discussing disagreements without conflict. Some relatives shared feelings of powerlessness when patients would not listen to them. Patients sometimes made (impactful) decisions for the sake of their loved ones. At the same time, some patients also spoke about their choices through the personal autonomy discourse and shared accounts of going against their relative’s preferences in making decisions.

When relatives talked about patients’ healthcare choices, they often spoke in terms of respecting the patients’ autonomy: “*I cannot make a choice for him. Nor do I want to*” [Mary, wife]. Relatives stressed that patients had a sound mind or that it concerned patients’ own body, so the choices should be theirs. Still, in their “doings,” these relatives tried to influence some of the choices the patient made. Mary talked about implicitly trying to convince her husband of accepting a lifesaving treatment he was reluctant about: “*I was certainly not prepared to bury him. We looked at him with big puppy dog eyes: please. So finally, he said: okay. At that point, I think he really did it for us.*”

Whilst relatives played a big role in patients’ lives and vice versa, relatives played a smaller role in interactions with HCPs. Relatives were mostly present at consultations in the hospital, yet patients often visited their general practitioners and allied health professionals—or incidentally all HCPs—by themselves. In the consultations, that relatives visited, they were often in the background and HCPs involved them only to a limited extent. In multiple consultations, HCPs made minimal eye contact with relatives, and seldomly directed questions explicitly at them. The possibility of eye contact between relatives and HCP was sometimes limited by (dual) computer monitors that blocked the relative’s view.

The roles that relatives took upon themselves within HCP interactions were asking questions, adding to what the patient had said, bringing forward their own worries to HCPs, and advocating on the patients’ behalf. For instance, Ralph challenged an HCP who wanted to wait at least 6 months before considering a surgery his wife desired due to pain: “*Why do you have to wait another six months for that?* [Addressing his wife] *Your way of living is becoming increasingly unpleasant. You can do less and less. You are increasingly in pain.*”

Some relatives shared feeling restricted in the questions they could ask HCPs, as they feared upsetting the patient with their questions or had explicit instructions from patients to not ask those questions. Sometimes relatives bypassed patients by sharing their worries with HCPs nonetheless to require medical attention to the areas they worried about. HCP 3’s reflection on such an expression of worry illustrates this: “*That really alarmed us and made us realize that there was more going on at home then he* [the patient] *was willing to tell us.*” In contrast, patients sometimes explicitly invited relatives in consultations to share their perspectives or questions.

When asked in interviews about the role of the relative, multiple HCPs could not comment on this: “*I must be completely honest and say that I cannot really remember what she was like during that conversation. In my experience, I mainly spoke to him*” [HCP15]. This lack of awareness of the relatives’ role was often explained in interviews by HCPs with the argument that the patients were capable of telling their own story and mentally competent for decisions. Some HCPs, however, did have some understanding of the perspective of the relative and made an effort to involve them in their care. These were mostly HCPs who had been involved with the patient for a longer time already or within an intensive care relationship, and general practitioners of whom relatives were also patients.

### The tension between navigating fragmented healthcare with interrelated conditions

#### Illustrative portrait: “I find it a bit upsetting, that you’re left alone with your illness”

Within the period of 6 months, Otis had more than 30 healthcare appointments with more than 18 HCPs and 4 invasive procedures taking place in three hospitals and two allied health professionals’ offices. Many of his healthcare providers limited their involvement to their own expertise: “*My colleague* [from the same specialty] *saw him for his back and legs. I mainly saw him for his arm, so that’s what I focused on. Besides that, I saw that he’s also seeing a surgeon, but I have no idea what that’s about. I keep completely out of that*” [HCP13]. Responsibilities were delineated: “*It’s not up to me to take that on myself. I refer him back to his general practitioner. My responsibility in that area actually ends there*” [HCP12]. Information transfer between the different hospitals was poor, which left Otis to be the messenger between HCPs. Some HCPs problematized this situation: “*It’s a shame we don’t have a letter from the surgeon stating ‘this is my plan and these are my concerns’. Because now it’s only been communicated through the patient. I find that difficult. I think Otis has a very clear understanding of his problems and is able to communicate that information well. But as a patient, you tend to hear only certain things that are said during conversation*” [HCP11]. But still, communication was mostly left to Otis: “*It seemed a good idea for him to inform the surgeon himself about the developments*” [HCP11]. The trust put in Otis was high: “*I haven’t checked his file to see exactly what the cardiologist wants, or the surgeon wants, but he can explain it to me. It sounds so reliable that I accept it as true, without combing through his entire file to see if it’s actually correct*” [HCP13]. However, sometimes Otis and his wife were struggling. Otis had difficulties keeping up with his different conditions: “*At first it was only the prostate cancer, but now four other things. It’s difficult to keep them apart, to learn about the diseases.*” They tried to get support from their general practitioner, but without success, as his wife shared: “*I do find it a bit upsetting that you’re then left on your own with your illness. You need someone to discuss it with, but the general practitioner didn’t know anything either.*” Otis initially had the expectation that his HCPs would have gotten together to coordinate his care: “*I don’t think it happened even once.*” When Otis turned to HCPs with his worries and questions, they referred him to their colleagues: “*I notice he is very preoccupied with this and very anxious about it. But that’s the part where I can’t help him because it’s not my area of expertise. So I try to point him in the direction of where he can get that information*” [HCP11]. Ultimately, Otis turned to me asking advice: “*What would you recommend your grandpa?*”

#### Description of tension

The interaction between patients’ multiple conditions and treatments with each other frequently made patients’ health a vulnerable balance. Multimorbidity led to conflicting health needs that patients and HCPs needed to manage and prioritize between, which could lead to complicated healthcare decisions.

*The heart, the fluid balance, it’s all interconnected. And that in turn affects the bladder. So, if on the one hand I’m working on the bladder, and other hand a diuretic is started because that’s necessary for the heart, then I can’t always fix everything.* [HCP24]

In dealing with their multiple conditions, patients were treated by multiple HCPs in different healthcare centers. These HCPs frequently needed to collaborate to inform their own treatment decisions. Subsequently, the patient trajectories showed many examples of HCPs seeking and giving each other advice or referring patients to colleagues. Whilst there were many examples of HCPs consulting each other, there were also many examples of HCPs who limited their involvement to their own expertise and did not feel responsible beyond their specialty. Frequently these HCPs deemed collaboration unnecessary: “*I think that my treatment doesn’t have that much influence on the overall situation*” [HCP 46]. Sometimes, however, other HCPs in those trajectories did find coordination necessary and were frustrated by their colleague’s lack of collaboration.

Many HCPs seemed to experience or enact a limited sense of responsibility for patients’ overall health. Care coordination, and by extension decision coordination, was often left to patients and relatives themselves. Patients and relatives frequently encountered a lack of collaboration and coordination between HCPs, alongside hassles, distress, contradictory messages, and health risks. An example of this was a near last minute operation cancellation due to omission of incorporating kidney disease into blood thinner regimens. Some patients, relatives, and HCPs explicitly wished for better care coordination, and regularly, patients, relatives, and sometimes even HCPs lost oversight of the situation.

A complicating factor in HCP collaboration and care coordination was the deficient information transfer within patient trajectories. Between different healthcare centers, exchange of patient file information—mostly through letters—was often untimely, required effort or lacked completely. Consequently, patients were frequently messengers relaying information between their various HCPs. Patients could not always relay information in the level of detail HCPs preferred and incidentally made mistakes if they did not fully grasp the situation, leading to miscommunications and information gaps for decision-making within the patient’s trajectory. A general practitioner shared frustration:

*I had to always ask the patient how things were going. Have you had that intervention yet, or is the cardiologist satisfied? Have they changed anything about your medication?* [That medication] *is much more delicate and often affects renal clearance, where I feel the monitoring needs to be much tighter*. [HCP35]

A few HCPs, mainly ones that had already been involved with patients for years, approached patients’ health(care) in a holistic way and articulated feeling responsible or took up responsibility within patients’ care or coordination beyond their medical expertise. These HCPs were often (highly) valued by patients.

## Discussion and implications

In this article, healthcare choices of older people with multimorbidity were studied from multiple perspectives in longitudinal patient trajectories through interviews and observations. These choices were in essence understood as ongoing, entangled, existential, and relational processes that are shaped by, yet also transcend healthcare contexts. The patients’ lifeworld, particularly their temporal, relational, and existential dimensions, and the relational and systemic contexts in which choices took place in healthcare institutions notably influenced these choices. Multiple tensions were found between these various elements shaping the patients’ healthcare choices.

One of the main findings of this study is the tension between patients not experiencing choice, whilst simultaneously enacting decisions. This finding points us towards the differences between what people say they do and what they actually do and therefore to the value of contextualizing interview data with observational data (O’Reilly, 2012; Slatman, 2014). At the same time, this finding demonstrates that apparently the experience of choice does not correlate one-to-one with making decisions in practice. Therefore, simply increasing the number of decisions patients can make within clinical contexts will probably increase their sense of choice only to a limited extent. This lack of resonance between the concept of choice and patients’ experiences of choice in healthcare in practice, specifically also in the end-of-life phase, has been discussed previously (Borgstrom, 2015; Drought & Koenig, 2002; Mol, 2008; Whitelaw & Clark, 2025).

Through combining interviews and observations, our study offers insight into *why* patients do not experience choice while they are enacting choice in practice. We found diverging experiences underlying such statements, ranging from more existential reasons such as experiencing the body, diseases and the future to be uncontrollable to more concrete reasons such as not overseeing one’s healthcare or not being given the option by HCPs or the healthcare system to make one’s preferred choice. This finding underscores the evasiveness of the concept of choice in healthcare and suggests that HCP should take these various underlying experiences into account when making decisions with patients. Such an exploration of these experiences may be useful in attuning care to specific patients, since a patient who doesn’t understand what their options are probably needs different types of support than a patient who feels compelled towards certain therapies due to the severity of their symptoms. Findings from conversation analysis studies suggest that HCPs’ phrasing also impacts patients’ sense of choice, so these methods may offer an avenue to further explore our observed misalignment (Pilnick, 2008; Stivers et al., 2018).

Our finding that the (bio)medical, technical perspective of HCPs overshadows the personal, existential perspectives of patients and relatives is especially relevant when considering the finding that HCPs made multiple decisions without involving patients in these decisions. Whilst this practice can be an integral part of good care (Bandini, 2020; Mol, 2008), it is important that these decisions are then informed jointly by the HCP’s professional expertise and the patient’s and relatives’ needs and value system and not the HCP’s. Unfortunately, the latter practice is not uncommon in healthcare (Rake et al., 2022; Spinnewijn et al., 2020). Our results question whether HCPs always have enough insight into the patients’ lifeworld to adequately inform HCP-driven healthcare decisions. This finding aligns with Sevenhuijsen (2003), who advocated for “moral communication” in which HCPs should explicitly explore their patients’ subjectivity and not mistakenly believe that they can understand their patients solely through empathy. This type of communication is particularly important when considering the common assumption of patients that physicians will be aware of and act in accordance with their personal values (Toombs, 1987).

Traditionally, shared decision-making models place more emphasis on information transfer about decision-making options than on eliciting patient perspectives (Bomhof-Roordink et al., 2019). Whilst some authors have suggested alternative approaches to (shared) healthcare decision-making that decenter the patient-HCP encounter and foreground patient context and relations (Clayman et al., 2017; Weiner, 2022), the existential dimension of healthcare decision-making is not sufficiently part of these models. In the “purposeful shared decision making” conceptualization of Hargraves et al. (2019) issues regarding meaning are explicitly part of one out of four “modes” of shared decision making. Although this is already an improvement to previous models, in the light of our findings, we would recommend issues regarding meaning to be part of all types of healthcare decision-making, as existential issues were found across all types of decisions.

Just as our findings show, the importance of relatives and their comparative disregard in healthcare settings at the end-of-life phase has previously been described (Sallnow et al., 2022). Our multiperspective design allowed us to illustrate the complexity of relatives’ involvement in more detail and to compare and contrast the accounts of patients and relatives, highlighting the (subtle) tensions between those. In addition, the combination of interviews and observations permitted us to show that relatives’ narratives centering patient autonomy were sometimes contrasted in practice by behaviors aimed at influencing patients’ opinions.

These findings further highlight the importance of relatives and the necessity to increase their involvement in healthcare. Many scholars have advocated for a more relational understanding of patient autonomy and care (Gómez-Vírseda et al., 2019; Mackenzie & Stoljar, 2000; Tronto, 1993), and specifically in the case of multimorbidity involvement of relatives has also been advocated (Kivelitz et al., 2021; Schulze et al., 2022). HCPs need the awareness that relatives play an important role on decision-making and that this should not be considered a threat to patient autonomy as relatives are naturally an important part of patients’ identity (van Nistelrooij et al., 2017). Our findings suggest that an exploration of relatives’ perspectives on decision-making would increase HCPs’ insight into what is at stake for relatives and patients and might enable HCPs to better support them both in decision-making processes.

Our results align with the work of other scholars who problematize the single-disease focus and fragmentation of the healthcare system in the light of the increasing incidence of multimorbidity (Boyd et al., 2019; Salisbury, 2012; Skou et al., 2022). Through closely following patient trajectories, our findings gave additional insights into the discrepancies between the accounts of different involved HCPs regarding the need for interprofessional collaboration. Whilst some HCPs felt that collaboration was not necessary, other HCPs felt in need of coordination with those HCPs, and problems occurred that could have been prevented through better collaboration or information transfer. This finding suggests that HCPs can have blind spots in their assessment of whether collaboration is necessary.

Furthermore, we found insufficient information transfer between different healthcare settings in combination with a limited sense or enactment of responsibility by many HCPs for patients’ healthcare as a whole. This created problems and healthcare risks and frequently put the burden of coordination on patients’ and relatives’ shoulders. Both these points offer important avenues for improving healthcare for older adults with multimorbidity. This implicates that further research and policy efforts are needed to find and examine solutions to enhance collaboration and deliver well-attuned, holistic care.

In the different trajectories, the types of and severity of multimorbidity-related problems encountered were diverse, indicating the heterogeneity of the group of patients with multimorbidity. Whilst some patients had multiple health conditions requiring (sometimes conflicting) interventions and close HCP collaboration during the study period, others had predominantly one “active” condition requiring attention, nonetheless within the constraints formed by their other more stable conditions and corresponding treatments. From the interviews where patients also shared previous experiences, it followed that throughout time the complexity of their multimorbidity was changeable. This changeability emphasizes that healthcare policy aimed at patients with multimorbidity needs to be adaptable to changes of healthcare needs over time. The heterogeneity within the multimorbidity population has led to criticism of quantity-of-conditions-based definitions of multimorbidity and advocation of more specific “complex multimorbidity” definitions as quantity-based definitions might not adequately reflect the severity of multimorbidity-related problems (Skou et al., 2022; World Health Organization, 2024). This argument is supported by the diversity of our population regarding these problems recruited with the quantitative inclusion criterion of ≥3 chronic conditions. Therefore, when using solely quantity-based definitions, policy makers should realize that a one-size-fits-all approach to this group will be far from effective. Finally, disease-related aspects formed only one part of the complexity of patients’ healthcare. When thinking about multimorbidity-related complexity, other personal, relational and systemic factors contributing to the complexity of patients’ situations should not be disregarded (Braspenning et al., 2025).

### Strengths and limitations

Our study had an intensive longitudinal and multiperspective design combining observations, interviews, and patient files as data sources. This design enabled us to get a very detailed and rich insight into patient trajectories of older adults with multimorbidity and the healthcare choices these patients, their relatives, and HCPs are faced with. This study answers the call for more nuanced evidence from longitudinal and multiperspective research in end-of-life topics (Kendall et al., 2009; Murray et al., 2009; Thölking et al., 2025) and for phenomenological research that contextualizes its findings (Slatman, 2014). Considering the intensity of the data collection and analysis of such a design and to achieve depth rather than breadth, the population was restricted to nine trajectories. To maximize the relevance of the results for a broader population, nonetheless, we strove for heterogeneity of the included trajectories (Dahlberg et al., 2008). We were successful in achieving diversity in sex, types of chronic conditions, types of involved relatives and HCPs, and education level. Our sampling strategy in which most HCPs were invited to participate in the study because their patient was already included in the study, mitigated potential selection bias that could occur if HCPs mainly nominate patients they get along well with for participation.

As is the case with most observation studies, we cannot rule out that HCPs acted differently in their interactions with patients than they normally would, exposing our findings to observation bias (Goodwin et al., 2017). On some occasions, patients expressed that HCPs took more time with them than they normally did, indicating that our findings may paint a too positive picture of the patient-HCP interactions. On the other hand, we hypothesize that there might also be a chance that HCP understand the studies’ focus on decision-making to be about good information transfer towards patients—as reflected by shared decision-making models (Bomhof-Roordink et al., 2019)—or might feel less free to discuss more existential topics with a researcher present. However, since there are examples of studies without observation to come to similar conclusions (Lilleheie et al., 2020), we assume the impact on our findings to be limited.

Finally, our results should be interpreted in the light of the Dutch context in which our data were collected. Since the Netherlands is a resource-rich healthcare setting in which essential primary and hospital care and basic home care are largely covered by mandatory basic insurance, financial considerations played only a marginal role in our findings. This finding is most likely not generalizable to healthcare systems with different financial contexts. Similarly, the influence of the medical gaze, the role of relatives, and ways in which health care systems are fragmented will differ across different national and cultural contexts. Therefore, the relevance of some or our results to other healthcare settings may be limited. Conducting similar research in different cultural and systemic contexts, would offer important insights into which aspects of our findings are transferable across settings and which are specific to the Dutch context.

## Conclusion

Older adults with multimorbidity are faced with a wide variety of healthcare choices, frequently with existential implications on their daily lives. Even though they make some decisions for themselves, patients don’t necessarily experience choice due to a wide range of underlying experiences ranging from practical to existential. Patients’ and relatives’ lives are often entangled putting relatives in a significant role regarding healthcare choices, which frequently results in tensions between both perspectives. However, just as for the existential dimension of healthcare choices, HCPs explore this relational aspect of the patients’ lifeworld only to a limited extent. Our results illustrate the importance of HCPs exploring these existential and relational perspectives to better inform decision-making and support for patients and relatives. The fragmented care patients receive in our single-disease focused healthcare system further complicates decision-making and often burdens patients and relatives with care coordination. The healthcare system needs change to facilitate more holistic, attuned care for this heterogenous population, particularly regarding information transfer and distribution of responsibility for coordination between HCPs and patients.

## Supplementary Material

gnag216_Supplementary_Data

## Data Availability

This study was preregistered at Open Science Framework [https://osf.io/xdvjs]. The participants of this study did not give written consent for their data to be shared publicly, so due to the sensitive nature of the research supporting data is not available.
