# Supplementary material for: “I don’t know whether I really have choices”: a multiperspective and longitudinal exploration of healthcare choices of older adults with multimorbidity
Source: Gerontologist. 2026 Sep 8;66(11):gnag216. doi: 10.1093/geront/gnag216 (PMC13661628; doi:10.1093/geront/gnag216)
Supplement: Supplementary file 1 [file gnag216_supplementary_data.pdf]

<u>Supplementary material title page</u>

Title article

"I don't know whether I really have choices": a multiperspective and longitudinal exploration of healthcare choices of older adults with multimorbidity

Authors

Thessa W. Thölking, MD, MA

Yvonne Engels, PhD

Yvonne Schoon, MD, PhD

Els J. van Wijngaarden, PhD

Contents

Supplementary 1 - Elaboration on methodology based on COREQ guidelines

Supplementary 2 - Details on observations

Supplementary 3 - Observation guide

Supplementary 4 - Interview topic guide with exemplary questions per theme per type of participant

Supplementary 5 - Details of interviews per patient trajectory

Supplementary 6 – Details per patient trajectory

Supplementary 7 – Overview of types of choices within patient trajectories

<u>Supplementary Material</u>

Supplementary 1 - Elaboration on methodology based on COREQ guidelines [Tong et al., 2007]

Domain 1: research team and reflexivity

1. Interviewer
   All data was collected by researcher TWT.
2. Credentials
   TWT: MD and MA
   YE: PhD
   YS: MD, PhD
   EVW: PhD
3. Occupation
   TWT: PhD-candidate
   YE: professor 'Sense of Meaning in Healthcare'
   YS: geriatrician and professor in Geriatrics
   EvW: associate professor in Meaning and Ethics at the End of Life
4. Gender
   All researchers are female
5. Experience and training
   TWT: previously involved in four qualitative and two quantitative research projects during her training in medicine and care ethics, of which three as primary researcher. Formally trained in qualitative research through a master's degree in care ethics with additional qualitative interview training.
   YE: considerable experience with qualitative research as a professor
   YS: was trained as a geriatrician and works still as a consultant in geriatrics, besides she supervised a lot of junior researchers, both in quantitative and qualitative research methods, nowadays as a professor
   EVW: extensive experience with qualitative research as an associate professor
6. Relationship established
   No previous relationship existed between any of the participants and data collecting researcher (TWT). A few participating healthcare professionals had prior relationships to researchers YE and YS due to their occupation at the same institution and previous collaboration. However, YE and YS did not have contact with those healthcare professionals about the study.
7. Participant knowledge of the interviewer
   Patients, relatives and healthcare professionals participating in the study were informed about the interviewer being a physician and care ethicist who was conducting the study as part of her PhD-research. When participants inquired, the interviewer explained that the research was driven by the wish to understand

how healthcare decisions of older patients with multimorbidity as this topic is understudied but deserves more attention due to its complexity and commonness. The information letter contained the information that the study team was based in the 'anesthesia, pain and palliative medicine' department, however participants were not informed of the special interest of the research on end-of-life issues. The only other reference to the end-of-life topic of the study was the 'surprise question' (see article for more details) recruiting physicians were asked to answer as part of the inclusion criteria.

8. <u>Interviewer characteristics</u>
   As the interviewer has a background in care ethics, her analytical insights are primed towards the critical insights at the core of this political ethic (e.g. relationality, contextuality, vulnerability, power and position, embodiment, meaning). Her research interest in the topic stems from a wider interest in ethical-existential questions regarding ageing and end-of-life in which she has previously done other research projects.

<u>Domain 2: study design</u>

9. <u>Methodological orientation and theory</u>
   The study was rooted in phenomenology and ethnography
10. <u>Sampling</u>
   Patients were selected through purposive sampling. Diversity was sought in sex, types of chronic diseases, different types of involved healthcare professionals, education level and socio-economic status. To enhance the variety in patient characteristics, patients were included from different settings (primary care and a university medical center). We approached general practitioners from different practices and university medical center specialists from different specialties to provide one patient each for participation. The final two included participants were provided by one healthcare professional, as this professional was able to provide participants from groups that were underrepresented in the study sample and had been difficult to recruit through previous efforts.
   Included patients were asked to invite one relative who was involved in decision making for contributing to interviews. If additional relatives were present at appointments with healthcare professionals, those were also invited to participate in the study. One of these 'additional' relatives offered to give an interview, therefore this relative was also included in interviewing. In one patient trajectory, a second relative was identified as relevant from interviews and therefore also invited for participation and interviewing. Healthcare professionals who had appointments with patients in the study period were invited to participate in the study.
11. <u>Method of approach</u>
   Patients were approached by their treating physician if they could be approached

by the researchers. Researcher TWT contacted all patients by telephone and informed them about the study. If patients were interested in participation, the formal information letter was sent by mail. After repeated telephone contact to confirm if patients were still willing to participate, a face-to-face meeting was scheduled at the patients' home to give patients the opportunity to meet the researcher and ask any additional questions about participation. One face-to-face meeting took place at the hospital before a healthcare appointment due to logistical challenges.

12. Sample size

    Nine patients participated, eleven relatives and 58 healthcare professionals.

13. Non-participation

    One patient was not included as they had a similar profile to another already included patient. Seven patients declined participation. Mostly without stating reasons. When reasons were stated, this was mostly the energy investment of participating in the study. One patient who visited their healthcare professionals alone did not want to burden their relatives with study participation. As they brought valuable diversity to the group, the patient was included without a relative. One patient died approximately halfway through the study period, after which the remaining interviews were held with closes ones and healthcare professionals.

14. Setting of data collection

    Observation data

    Appointments with healthcare professionals took place in various settings, including patients' homes, primary care clinics, allied healthcare professional offices, healthcare centers, hospital out-patient department in regional hospitals, a specialized hospital and a university medical center, and clinical wards.

    Interviews

    Face-to-face interviews with patients and relatives were held at participants' homes, except for two interviews that took place in the hospital. Face-to-face interviews with healthcare professionals took place in their workplace.

15. Presence of non-participants

    All interviews were one-on-one interviews between the researcher and the participant. Except for incidental and short disruptions by others (participants and non-participants alike) during interviews, no one else was present during interviews.

16. Description of sample

    See table one in the manuscript

17. Interview and observation guide

    See supplementary 3 and 4

18. Repeat interviews
    Repeat interviews were held with all patients, with seven relatives and three healthcare professionals.
19. Audio/visual recording
    Interviews and observations of healthcare appointments were audio recorded. Five observations were not audio recorded. For one patient, some observations took place on a clinical ward with other patients present in the room. These observations were not audio recorded and only documented by written notes. One observation was not audio recorded as requesting permission to start the recording was estimated to disrupt the integrity of the interaction between the patient and healthcare professional.
20. Field notes
    Observations
    Fieldnotes were during researcher interactions with patients and relatives whilst waiting for healthcare professional appointments and during the appointment itself. Fieldnotes were completed after conclusion of the observation.
    Interviews
    Staccato fieldnotes were made during the interviews, and more elaborate fieldnotes were made after the conclusion of the interviews.
21. Duration
    Observation
    A total of 31 hours of observation was audio-recorded. Additional observation time before and after appointments and of the five non-recorded appointments was not recorded.
    Interviews
    A total 55 hours of interviews was audio-recorded. Patient interview mean duration was 77 minutes (range 31-105), relative interview mean duration 59 minutes (range 17-119) and healthcare professional interview duration 30 minutes (range 16-62).
22. Data saturation
    As this study was conducted with a phenomenological approach, data saturation was not discussed
23. Transcripts returned
    Transcripts were not returned to participants.

Domain 3: analysis and findings

24. Numbers of data coders
    Two patient trajectory narratives were co-constructed by SVF and TWT, after which TWT finished the remaining narratives. YE and TWT coded the longest narrative independently and discussed their findings. TWT coded the remaining narratives.

25. Description of the coding tree
    As coding is only a tool within phenomenological analysis to sort and organize meaning units, there is no description of a coding tree
26. Derivation of themes
    Themes were identified inductively from the data
27. Software
    Atlas.ti (version 24) was used for coding
28. Participants checking
    Patients and relatives did not give feedback on the findings. However, a sound board meeting was held in a community center for older people to understand how the findings resonate in a group of older people with experiences as patients and relatives. Three participating healthcare professionals were invited to a similar sound board meeting with the same purpose.
29. Quotations presented
    The themes are illustrated by quotations. Quotations are provided with participant identifiers.
30. Data and findings consistent
    In reporting, we aimed for consistency between the themes and the quotations used to support these findings.
31. Clarity of major themes
    In reporting, we aimed for clarity in the description of the major themes
32. Clarity of minor themes
    In reporting, we aimed to report diverse cases and pay attention to exception cases.

Reference

Tong, A., Sainsbury, P., & Craig, J. (2007). Consolidated criteria for reporting qualitative research (COREQ): a 32-item checklist for interviews and focus groups. *International Journal for Quality in Health Care*, *19*(6), 349–357. https://doi.org/10.1093/intqhc/mzm042

Supplementary 2 - Details on observations

The aim of the researcher was to observe as many healthcare appointments of patients as possible. The focus was on appointments in which healthcare choices and conversation between the patient (potential relatives) and healthcare professionals were expected to be (significant) part of the consultation. Therefore, appointments were not observed if their sole purpose was performing a diagnostic or treatment procedure. If an appointment with a healthcare professional was observed and it was not deemed relevant regarding healthcare choices, subsequent appointments were not visited, unless there was reason to expect them to be relevant going forward. If patients had very frequent contact with healthcare professionals (e.g. during hospitalization or institutional living situation), one or a few significant moments were chosen to observe (e.g. weekly ward rounds or family information meetings). If patient appointments coincided, either the appointment that was expected to be most relevant regarding healthcare choices, or the appointment of the patient of whom less appointments had been observed was chosen. To assess the relevance of a certain appointment or to decide between conflicting appointments, the data collecting researcher (TWT) discussed her considerations with her daily supervisor (EVW). If the researcher could not attend a consultation and the healthcare professional approved, the patient and relatives were invited – without obligation – to record the consultation and provide the researcher with the recording. Incidentally, a healthcare professional was asked to record a consultation.

Patients and relatives were the ones to inform the researcher that appointments took place. Incidentally, an included healthcare professional would reach out to the researcher. The researcher had regular contact with patients between appointments to hear whether new appointments had come up. Patients were encouraged to inform the researcher about last-minute appointments, however, patients frequently forgot to inform the researcher, and sometimes prior commitments made it difficult to attend.

Supplementary 3 - Observation guide

Observation of interactions between healthcare professionals, patients, and patients' relatives, with a special interest on choices and end-of life issues.

0. Place, date, situation, time
1. Initial impressions:
    a. Key components of the observed interaction
    b. (Detailed) sensory information about
        i. Physical setting (sketch)
            1. Positioning of participants
            2. Furniture/design
            3. Striking items and/or attributes
            4. Sounds/colours/temperature/smell
        ii. Involved participants
            1. Appearance
            2. Striking features
            3. Posture/attitude
            4. Impression
2. What is significant or unexpected?
    a. Incidents
    b. Emotions
    c. Impressions
    d. (non)verbal interactions
3. What seems to be significant or important to the participants?
    a. When do they stop/decrease or increase volume/change eye contact?
    b. What do they talk about?
    c. What produces (strong) emotional responses?
4. How routine actions in the setting are organized and take place?
    a. Attention on (automatic) social and interactional processes
5. Broad impressions (with help of the lifeworld dimensions)
    a. Temporality
        i. Experience of available 'time' in interaction
        ii. Divergence in 'pace' between participants
        iii. Amount of time 'taken up' by various participants
    b. Spatiality
        i. Impact of the 'space' on the interaction
    c. Intersubjectivity
        i. Nature of the relationship between the participants
    d. Embodiment
        i. The body as object of interaction or examination
        ii. Expression of experience through the body
    e. Mood
        i. (Various) mood(s) experienced throughout the interaction

References

Emerson, R. M., Fretz, R. I., & Shaw, L. L. (2011). *Writing Ethnographic Fieldnotes* (Second ed.). University of Chicago Press.

Hemingway, A. (2011). Lifeworld-led care: Is it relevant for well-being and the fifth wave of public health action? *International Journal of Qualitative Studies on Health and Well-being, 6*(4), 10364. https://doi.org/10.3402/qhw.v6i4.10364

Supplementary 4 - Interview topic guide with exemplary questions per theme per type of participant

Actual formulation of interview questions during the interviews depended on the way participants naturally spoke about choices in a healthcare context (as questions were tailored to the language they used to express their experiences) and on the content of the study observations. Repeat interviews focused on developments in the various topics through time.

Topic 1: Interactions regarding choices in a healthcare context

- Patient: Can you give an example of a choice you recently made? Or that was made with or for you?
- Relative: Can you give an example of a choice that was recently made by or for [patient x]? How where you involved in this choice?
- Healthcare professional: Can you give an example of a choice you recently made with or for [patient x]?

Topic 2: Reflection on previously observed interactions between patients, relatives, and healthcare professionals

- Patient/relative: I accompanied you to appointment [x] with [healthcare professional x]. How did you experience this interaction? How did you experience choice-making within this interaction?
- Healthcare professional: I sat in on appointment [x] with [patient x]. How did you experience this interaction? How did you experience choice-making within this interaction?

Topic 3: End-of-life (only discussed if brought up by participant)

- You just mentioned [x] *(end-of-life/limited life expectancy/vulnerable health status/frailty/death)*.
    - Patient: To what extent does this play a role in the healthcare choices you make?
    - Relative: To what extent does this play a role in the healthcare choices of [patient x]?
    - Healthcare professional: to what extent does this play a role in choice-making with or for [patient x]?

  Can you give an example of this?

Topic 4: Working within a wider healthcare context (only for healthcare professionals)

- Can you describe in which way you collaborate with other healthcare professionals regarding [patient x]'s care? To what extent does this influence the choices you make with or for [patient x]?
- To what extent does your institution or your professional organization influence the way in which you make decisions with or for [patient x]?

Probing questions

- Can you describe this experience in as much detail as possible?
- Can you describe this experience in a way that I can imagine how it felt to have experienced it: what was said, what did you feel, what did you think, what was de mood?
- What do you mean when you say [x]?
- How do you experience [x]?
- Can you remember a choice that you made in your last appointment?
- You just told me about a positive/negative experience, have you also experienced the opposite?

Supplementary 5 - Details of interviews per patient trajectory[a]

Patient trajectory	Patient	Relative	Healthcare professionals
Trajectory 1	Initial interview and interview during study period due to major health event. No concluding interview due to patient's death.	Initial and concluding interview with partner	Telephone interview during study period with cardiologist
			Concluding interview with dialysis physician assistant
		Concluding interview with child	Concluding interview with dialysis nurse
			Concluding interview with nephrologist
Trajectory 2	Initial and concluding interview	Initial and concluding interview with partner	Interview during study period and concluding telephone interview with exercise therapist
			Interview during study period and concluding interview with cardiology nurse practitioner
			Interview during study period and concluding interview with neurosurgeon
			Interview during study period with neurology resident
			Concluding video-call interview with urologist (combined with interview of trajectory 7)
Trajectory 3	Initial and concluding interview	Initial and concluding telephone	Interview during study period with physiotherapist

		interview with partner	Video-call interview during study period with nursing associate
			Concluding interview with psychiatrist
			Concluding telephone interview with general practitioner
Trajectory 4	Initial and concluding interview	Initial and concluding interview with partner	Interview during study period with cardiologist
			Concluding video-call interview with general practitioner
			Concluding interview with pulmonologist
Trajectory 5	Initial and concluding interview	Initial telephone and concluding telephone interview with child	Concluding video-call interview with general practitioner
			Concluding video-call interview with home care nurse
			Concluding video-call interview with physiotherapist
			Concluding video-call interview with palliative care nurse
			Concluding video-call interview with informal care support coordinator
Trajectory 6	Initial and concluding interview	Initial and concluding interview with partner	Concluding interview with gynecology resident
			Concluding interview with rheumatology resident

Trajectory 7	Initial and concluding interview	Initial and concluding interview with partner	Concluding video-call interview with cardiology nurse
			Concluding phone interview with general practitioner
			Concluding video-call interview with urologist (combined with interview of trajectory 2)
Trajectory 8	Initial and concluding interview	n.a.	Concluding video-call interview with general practitioner (combined with interview of trajectory 9)
Trajectory 9	Initial and concluding interview	Concluding telephone interview with child	Concluding video-call interview with general practitioner (combined with interview of trajectory 8)
		Concluding telephone interview with child	Concluding video-call interview with dermatologist

[a]*Face-to-face interviews unless specified otherwise*

Supplementary 6 – Details per patient trajectory

**Trajectory number, place of recruitment & patient's age, sex and health conditions**[a]	**Included relatives**	**Nr. of included HCP**[b]	**Nr. and type of observations + type of HCP involved**	**General overview of events during study period**
1 – nephrology.<br><br>Male, 87 years.<br><br>Kidney failure, diabetes, coronary artery disease, atrial fibrillation, diabetic retinopathy, and restless legs syndrome.	Wife, daughter and son	10	1 outpatient consultation:<br>- cardiologist<br>1 allied health professional visit:<br>- medical foot care professional<br>4 ward round during dialysis:<br>- dialysis physician assistant<br>- 3 dialysis nurses<br>- nephrologist<br>1 family meeting during hospitalization:<br>- cardiologist<br>- cardiology nurse<br>- cardiology resident<br>1 bereavement family meeting:<br>- dialysis physician assistant<br>- nephrologist	At the beginning of the study, the patient was dealing with increasing cardiac symptoms for which cardiac consultation was sought, and the patient decided between two courses of action for treatment. Simultaneously, due to increasing difficulties at dialysis, increasing dialysis frequency and the use of compression stockings – requiring daily home care assistance – were discussed with the patient. After a fall and ankle fracture requiring operative fixation, the patient was hospitalized. A subsequent cardiac decompensation destabilized the patient's condition and further complicated dialysis. The patient opted for treatment at the cardiac care unit, disregarding doubts expressed by a cardiologist about the relevance of treatment. After the patient's cardiac situation stabilized, a family meeting was held to discuss invasive cardiac treatment, which the patient declined for the time being. After being discharged to the rehabilitation ward, the patient suffered a bleeding complication after which the patient died within 24 hours. The treating dialysis team held a bereavement meeting with the family to look back on the dialysis period and discuss any remaining questions of the family.

2 –cardiology.<br><br>Male, 78 years.<br><br>Heart failure, atrial fibrillation, cervical and lumbar spinal cord stenosis, chronic pain, prostate cancer, gout, osteoarthritis, aortic ectasia, and hypercholesterolemia.	Wife	18	13 outpatient consultations:<br>- anesthesiologist<br>- anesthesiology resident<br>- cardiologist<br>- cardiology nurse practitioner<br>- 2 neurologists<br>- 2 neurology residents<br>- neurosurgeon<br>- neurosurgery resident<br>- oncological urologist<br>- surgery resident<br>- urologist<br><br>9 telephone consultations:<br>- anesthesiologist<br>- cardiology nurse practitioner<br>- general practitioner<br>- neurology resident<br>- neurosurgeon<br>- pain medicine nurse<br><br>8 allied health professional visits:<br>- exercise therapist<br>- podiatrist	At the beginning of the study, the patient needed a pacemaker for heart failure and was being assessed for neurosurgical intervention for lumbar spinal stenosis. Whilst the cardiologist preferred the spine surgery to take place before the pacemaker implantation, the neurosurgeon decided not to operate as they saw little benefit of the operation, especially in relation to the patient's cardiac condition. Only after the patient had communicated this decision to his cardiac team, the pacemaker implantation was brought forward. However, this intervention was later postponed last minute as the patient was not able to lie still on his back due to pain from a cervical stenosis. The patient opted for an invasive pain treatment, after which the pacemaker implantation was scheduled. However, due to neurological deterioration, the neurosurgeon decided to operate on the lumbar spine anyway, before the pacemaker implantation. The pacemaker included an implantable defibrillator the patient opted for. Due to increasing neurological deterioration resulting from the cervical spinal stenosis after the back surgery, the neurosurgeon strongly recommended neck surgery, which the patient complied with. Simultaneously, the neurologist suggested an injection for carpal tunnel syndrome and the patient complied. Alongside these interventions, the patient had exercise therapy and (follow-up) consultations for his other conditions.

3 – psychiatry.<br><br>Male, 75 years.<br><br>Heart failure, hypertension, peripheral arterial disease, bipolar disorder, urine incontinence, and osteoarthritis.	Wife	4	1 allied health professional visit:<br>- physiotherapist<br>1 observation of daily care:<br>- nursing associate<br>1 telephone consultation:<br>- psychiatrist	The patient had been living in a long-term care facility for two years at the beginning of the study. He received a lot of support in his activities of daily living and household from his wife. During the study period, the health of the patient's wife deteriorated, and she was no longer able to visit and fulfill her normal activities. Therefore, the patient needed to find new ways of getting things done. This change was discussed in a psychiatric consultation. Somatically, the patient was relatively stable during the study and apart from his physiotherapy - of which one session was observed – and a few ad hoc consultations with the general practitioner that could not be observed, no somatic healthcare appointments took place. To get a sense of the daily care interactions within his care facility, one care moment was observed.
4 – pulmonology<br><br>Male, 80 years.<br><br>Chronic obstructive pulmonary disease, atrial flutter, kyphoscoliosis, osteoarthritis, osteoporosis, and hypercholesterolemia.	Wife	6	6 outpatient consultations:<br>- cardiologist<br>- general practitioner<br>- general practitioner assistant<br>- pulmonologist<br>1 telephone consultation:<br>- cardiologist<br>1 allied health professional visit:<br>- 2 physiotherapists	At the beginning of the study, the patient's cardiologist was discussing non-invasive treatment options with him for his atrial flutter, as he had recently declined an invasive treatment option. The patient followed the cardiologist's medication suggestion, which led to the stabilization of the patient's cardiac condition. Subsequently, however, the patient's pulmonary condition worsened for which the patient had multiple consultations with his general practitioner and pulmonologist who both tried medications to improve his situation. Additionally, the patient had meetings with his general practitioner to try to taper off his sleeping

				medication, as suggested by the general practitioner.
5 – general practice<br><br>Female, 92 years.<br><br>Heart failure, hypertension, peripheral neuropathy, restless legs syndrome, urine incontinence, and hypercholesterolemia.	Daughter	6	<u>5 home visits:</u><br>- coordinator of informal care<br>- general practitioner<br>- general practitioner assistant<br>- 2 home care nurses<br>- physiotherapist	At the beginning of the study, the patient started with physiotherapy to improve her mobility after experiencing increasing mobility issues and falls. However, after a few sessions, the patient had growing doubts whether the physiotherapy had purpose or whether her physical decline was inevitable. She eventually decided to discontinue the therapy, even though the physiotherapist had encouraged her to continue. After purchasing a few mobility aids, the patient realized that it was the question whether she could still live independently. Therefore, she started visiting long-term care facilities throughout the country. To aid her in her decision between moving to a care facility and staying home with further home adaptations, a meeting was planned with the patient's home care nurse, a specialist palliative care home care nurse and the patient's informal care support coordinator in which the topic was discussed. By the end of the study, the patient decided to postpone the decision about housing, as she had difficulties overseeing it. Alongside her mobility issues, the patient had her annual physical check-up, and she was seen by her general practitioner for increasing cardiac symptoms.
6 – gynecology<br><br>Female, 77 years.	Husband	4	<u>6 outpatient consultations:</u><br>- gynecology resident<br>- orthopedics physician	During the study the patient had multiple gynecology consultations for her pelvic organ prolapse, trialing different pessary to find a suitable

Polyarthrosis with multiple joint replacements, chronic pain, pelvic organ prolapse, polymyalgia rheumatica, chronic constipation, thrombotic disorder, hemochromatosis, hypertension, osteoporosis, and xerosis.			assistant<br>- rheumatology resident<br>- surgery nurse	one, as she had previously declined surgery. The patient visited a surgery nurse to discuss therapeutic options for her chronic constipation that contributed to her prolapse after trying various medication options that were not satisfactory to the patient. The surgical nurse outlined various lifestyle changes from which the patient chose a few to implement. Due to hip pain, the patient requested hip replacement surgery, however her osteoarthritis was not considered advanced enough to qualify for the surgery. As time went on, the patient experienced increasing mobility issues which turned out to be caused by polymyalgia rheumatica, for which medication treatment protocol was followed.
7 – general practice<br><br>Female, 81 years.<br><br>Heart failure, hypertension, cardiac arrhythmia, chronic pain, urine incontinence, asthma, rheumatic disorder, glaucoma, and eczema.	Husband	5	<u>3 outpatient consultations:</u><br>- cardiologist<br>- cardiology nurse<br>- general practitioner<br><u>2 telephone consultations:</u><br>- pain medicine nurse<br>- urologist	After a year of serious health problems, the patient's health was relatively stable during the study period. She had follow-up appointments within the cardiology and urology department and had a urinary tract infection that was treated by the urologist. In addition, she had an invasive pain treatment that caused some cardiological and gastro-intestinal symptoms.
8 – general practice	None	2	<u>1 outpatient consultation:</u><br>- general practitioner	During the study period, the patient's health was relatively stable. The patient experienced shoulder

Male, 80 years.<br><br>Cardiac arrhythmia, hypertension, hypercholesterolemia, partial deafness, insomnia, and anemia.			<u>1 allied health professional visit:</u><br>- physiotherapist	pain, for which he sought consultation with his general practitioner who suggested physiotherapy. The patient went along with this suggestion; however, he decided to discontinue the physiotherapy after a few sessions since he felt he experienced no benefit from the sessions.
9 – general practice<br><br>Female, 82 years.<br><br>Asthma, coronary artery disease, hypertension, renal insufficiency, skin cancer, partial deafness, thyroid cyst, hay fever, and dust mite allergy.	Daughter and son	5	<u>6 outpatient consultations:</u><br>- dermatologist<br>- general practitioner assistant<br>- 2 general practitioners<br>- pulmonology nurse	At the beginning of the study period, the patient was recovering from serious illness. In consultation with her general practitioner, she decided to give medication a try for her decreased kidney function, which was followed up by the general practitioner. The patient had follow-up consultations for her pulmonary disease. Unsure who to contact about a skin lesion that had previously been subject to dermatological and plastic surgery intervention, the patient visited her general practitioner. Her general practitioner asked for a second opinion from his colleague, who in turn found out that the patient should have been followed up by the dermatology department but was never called in. At the subsequent dermatological consult, the dermatologist suggested watchful waiting after their initial suggestion of removal of the lesion by a plastic surgeon was met by hesitation of the patient. The patient opted for watchful waiting, only to revisit her decision after a few days and to opt for the removal. Towards the end of the study period the patient contacted the general practitioner

				because of gastro-intestinal symptoms, which turned out to be caused by metastasized cholangiocarcinoma.

[a]*As reported by the patient at the start of study. Incidentally, additional conditions were added by the researcher if they were prominent during the observations (supported by patient files) or developed during the study.*

[b]*Two healthcare professionals were part of two patient trajectories*

Supplementary 7 – Overview of types of choices within patient trajectories

**Category**	**Examples**
Care	Support for activities of daily living, change of living situation, drafting residential care plans.
Lifestyle	Adherence to diet and exercise.
Mobility	Use of mobility aids, adherence to safety advice.
Access to healthcare	Seeking (extra) care and healthcare appointments, moving up appointments, choice of healthcare provider.
Patient-HCP relationship	Patient giving feedback to HCPs about how they want to be treated, filing complaints.
Diagnostics	Requesting or consenting to (invasive) diagnostic procedures.
Treatment	Decisions regarding medication, invasive procedures/surgeries, seeking treatment by allied health providers.
Treatment limitations	Refraining from or stopping treatment, discussing or drafting advance directives or living wills about treatment or euthanasia.
